# Informing HIV Prevention Programs for Adolescent Girls and Young Women: A Modified Approach to Programmatic Mapping and Key Population Size Estimation

**DOI:** 10.2196/11196

**Published:** 2019-04-01

**Authors:** Eve Cheuk, Shajy Isac, Helgar Musyoki, Michael Pickles, Parinita Bhattacharjee, Peter Gichangi, Robert Lorway, Sharmistha Mishra, James Blanchard, Marissa Becker

**Affiliations:** 1 Centre for Global Public Health Rady Faculty of Health Sciences University of Manitoba Winnipeg, MB Canada; 2 India Health Action Trust Bangalore India; 3 National AIDS and Sexually Transmitted Infection Control Programme Ministry of Health Nairobi Kenya; 4 Partners for Health and Development in Africa Nairobi Kenya; 5 International Centre for Reproductive Health Kenya Mombasa Kenya; 6 Li Ka Shing Knowledge Institute St. Michael’s Hospital Toronto, ON Canada; 7 Department of Medicine University of Toronto Toronto, ON Canada

**Keywords:** programmatic mapping, key population size estimation, female sex workers, adolescent girls and young women, sex work, transactional sex, casual sex, HIV prevention

## Abstract

**Background:**

Standard programmatic mapping involves identifying locations where key populations meet, profiling of these locations (hotspots), and estimating the key population size. Information gained from this method has been used for HIV programming—resource allocation, program planning, service delivery, and monitoring and evaluation—for people who inject drugs, men who have sex with men, and female sex workers (FSWs). With an increasing focus on adolescent girls and young women (AGYW) as a priority population for HIV prevention, programs need to know the location of and how to effectively reach individuals who are at increased risk for HIV but were conventionally considered part of the general population. We hypothesize that AGYW who engage in transactional and casual sex also congregate at sex work hotspots to meet sex partners. Therefore, we adapted the standard programmatic mapping approach to understand the geographic distribution and population size of AGYW at increased HIV risk in Mombasa County, Kenya.

**Objectives:**

The objectives are several-fold: (1) detail and compare the modified programmatic mapping approach used in this study to the standard approach, (2) estimate the number of young FSWs, (3) estimate the number of AGYW who congregate in sex work hotspots to meet sex partners other than clients, (4) estimate the overlap in sexual network in hotspots, (5) describe the distribution of sex work hotspots across Mombasa and its four subcounties, and (6) compare the distribution of hotspots that were known to the local HIV prevention program prior to this study and those newly identified.

**Methods:**

The standard programmatic mapping approach was modified to estimate the population of young women aged 14 to 24 years who visit sex work hotspots in Mombasa to meet partners for commercial, transactional, and casual sex.

**Results:**

We estimated that there were 11,777 FSWs (range 9265 to 14,290) in Mombasa in 2014 among whom 6127 (52.02%) were 14 to 24 years old. The population estimates for women aged 14 to 24 years who engaged in transactional and casual sex and congregated at the hotspots were 5348 (range 4185 to 6510) and 4160 (range 3194 to 5125), respectively. Of the 1025 validated sex work hotspots, 870 (84.88%) were locations also visited by women engaged in transactional and casual sex. Only 47 (4.58%) hotspots were exclusive sex work locations. The geographic and typological distribution of hotspots were significantly different between the four subcounties (*P*<.001). Of the 1025 hotspots, 419 (40.88%) were already known to the local HIV prevention program and 606 (59.12%) were newly identified.

**Conclusions:**

Using the adapted programmatic mapping approach detailed in this study, our results show that HIV prevention programs tailored to AGYW can focus delivery of their interventions to sex work hotspots to reach subgroups that may be at increased risk for HIV.

## Introduction

Key populations are groups of individuals who are at increased risk for, or who are disproportionately burdened by, a particular disease. Therefore, it is important for individuals of a key population to have access to necessary prevention resources, care, and support. In the context of HIV, key populations include people living with HIV, people who inject drugs, men who have sex with men, transgender people, and sex workers [1]. One of the earliest steps in designing a focused HIV program for a key population is knowing who and how many within a key population are at risk, where they are at risk, and what puts them at risk (eg, condomless sex, experience of violence, needle sharing). Various methods exist for estimating key population size such as capture-recapture, multiplier, population survey, and network scale-up [2,3]. However, these approaches only provide an estimate for the size of a key population, which in itself may not be sufficient for comprehensive program design, planning, and delivery. In recognition of the importance of how place, environment, and sexual network formation contribute to the epidemiology of HIV [4-6], programmatic mapping was developed to shed light on the types (or typology) and distribution of locations where key populations congregate. Programmatic mapping involves locating and profiling geographically defined locations (or hotspots) where key populations meet and risk behaviors take place and estimating the size of key populations in each hotspot. Individual hotspot-level estimates can be aggregated to generate population size estimates for a district, region, and entire country. Many cities and countries, including Kenya, have used programmatic mapping to determine the geographic distribution and population size for people who use injection drugs; men who have sex with men; and male, female, and transgender sex workers [5,7,8].

Given the disproportionately high burden of HIV shouldered by female sex workers (FSWs) [9], many HIV prevention programs have been implemented to provide focused services to this key population, and there have been successes in reducing new HIV infections among FSWs in some regions of the world [10-12]. Nonetheless, in a clinic-based survey of Kenyan FSWs between 18 and 57 years, HIV prevalence was highest among women who had been in sex work for less than two years [13], highlighting a program gap in addressing HIV risk early. In fact, this high HIV prevalence may indeed signal existing HIV risk factors prior to entry into sex work that remain unmitigated and that are likely compounded by other factors in the early stages of a sex work career.

Conventional FSW programs are not designed to reach women who engage in other types of sexual partnerships that may also be associated with increased risk of HIV acquisition—namely, condomless sex in the context of transactional sex and casual sex [14]. Adolescent girls and young women (AGYW) aged 15 to 24 years are particularly burdened by HIV. In 2016 there were an estimated 790,000 (range 680,000 to 910,000) new HIV infections among adult women globally, among whom 46% were AGYW. AGYW also represented approximately 60% of newly infected young people and of all young people living with HIV globally [15]. In Kenya, 32.8% of all new adult HIV infection in 2015 occurred among AGYW [16].

The shared vulnerabilities among AGYW associated with sexual debut and their early sexual experiences and among young FSWs who have newly entered sex work have led to a global call to refocus and Fast-Track HIV prevention among AGYW [17]. But how does one expand coverage of targeted HIV prevention programs to a subpopulation that has traditionally been regarded as part of the general population?

AGYW are a heterogeneous population. We hypothesize that AGYW who engage in transactional and casual sex and who congregate at sex work hotspots to specifically meet sex partners have higher vulnerabilities and are at an increased risk for HIV compared to their counterparts in the general population. Therefore, understanding whether and to what extent potential overlap in sexual networks exists between "high-risk" AGYW and FSWs could provide valuable information for prevention programs to reach and provide services for AGYW subgroups in the relevant settings. In this paper, we describe how we modified the current approach to FSW programmatic mapping [5,7,8] to gather information on the geographic distribution and estimate the population size of AGYW congregating at established sex work hotspots by the spectrum of sexual partnerships, including sex work and transactional and casual sex. The objectives of our research were several-fold:

- Detail and compare the modified programmatic mapping approach used in this study to the standard approach
- Estimate the number of young FSWs
- Estimate the number of AGYW who congregate in sex work hotspots to meet sex partners other than clients
- Estimate the overlap in sexual network in hotspots
- Describe the distribution of sex work hotspots across Mombasa and its four subcounties
- Compare the distribution of hotspots that were known to the local HIV prevention program prior to this study with those newly identified

Finally, we also reviewed how this modified approach to programmatic mapping and population size estimation could provide strategic guidance for HIV/sexually transmitted infection (STI) prevention programs looking to adapt and expand their services to address a wider spectrum of risk among AGYW within sex work hotspots.

## Methods

### Study Setting

Mombasa County is the smallest of 47 counties in Kenya, covering a territory of 294.9 km^2^, situated along the coast of the Indian Ocean [18]. In 2014, Mombasa had a population of 1,106,444 (with 319,032 women of reproductive age [14 to 44 years] and 134,885 AGYW [14 to 24 years]) [18]. Mombasa city is an important regional economic hub with a robust tourism sector and a port that handles millions of tons of cargo annually [19]. Kenya has a mixed HIV epidemic, and in 2015 the HIV prevalence in Mombasa County was 7.9% [20], 1.3 times higher than the national average of 5.9% [16]. The HIV prevalence among FSWs in Kenya was 29.3% in 2015 compared with 6.3% among women in the general population [16]. The HIV prevalence among FSWs in Mombasa is currently unknown. In 2014, HIV prevalence was estimated at 6.0% and 10.0% among AGYW in the age groups 15 to 19 years and 20 to 24 years, respectively [21].

### Definitions

Sex work is defined as the exchange of money (or gifts or other resources) between individuals for sex, whereby the negotiation of the price of sex is explicit between the sex worker and the client before any exchange takes place. Transactional sex is a more nuanced exchange between individuals who engage in sex with the expectation of receiving money, gifts, or other resources in return; however, the price of sex is not prenegotiated [22,23]. Casual sex occurs when individuals engage in sex but neither party expects to receive money, gifts, or other resources in return. A hotspot is defined as an indoor or outdoor venue or location where FSWs congregate to solicit clients and/or where sex work–related sexual activities take place.

### Standard Approach to Programmatic Mapping and Estimation of Key Population Size

#### Overview

**Figure 1:**
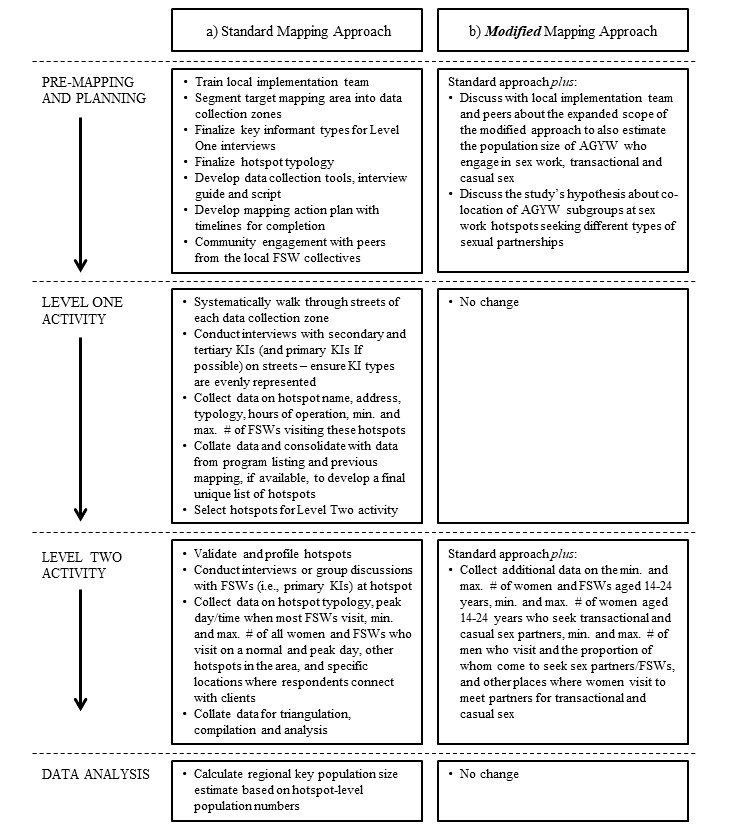
Comparison of the standard programmatic mapping approach with the modified approach used in this study. FSW: female sex worker; AGYW: adolescent girls and young women; KI: key informant.

The standard mapping method [5,7,8] (Figure 1) involves (1) premapping and planning exercise, (2) level one secondary and tertiary key informant (KI) interviews to generate an exhaustive list of hotspots where members of a key population congregate, (3) level two interviews (or group discussion) with primary KIs on site to validate and profile hotspots, and (4) data analysis. Primary KIs are members of the key population (in this case, FSWs), secondary KIs include persons who have close association with and an intimate knowledge about sex work (eg, pimps, brothel owners), and tertiary KIs include those who have a professional knowledge and/or interest in sex work (eg, taxi drivers, club bouncers, police).

#### Premapping and Planning Exercise

In the first stage of the mapping exercise, the local team of program implementers and peers—former or current members of the sex worker community—are trained and prepared for data collection. The local team discusses and finalizes a list of potential types of secondary and tertiary KIs for the level one interviews and the most common typologies of hotspots where members of the key population congregate. The team then segments the city into divisions within which smaller data collection zones are delineated, often based on existing administrative units, and develops an action plan with timelines. Community engagement with peers from local FSW collectives and nongovernmental organizations (NGOs) serving FSWs is a pivotal part of the premapping exercise. The study team needs to work with peers and NGOs because they hold invaluable knowledge about the sex work context and surroundings that an outsider does not have. More importantly, their involvement will ensure development of a mapping process that is well informed, socially and ethically responsible, and acceptable to members of the key population.

#### Level One Mapping Activity

The second stage of the mapping exercise involves systematically walking through the streets and open areas (eg, parks, market) of each data collection zone and interviewing secondary and tertiary KIs (and primary KIs as well if possible) as they are being approached by the local team in these public spaces with the aim to identify and generate an exhaustive list of hotspots by names and address. Through this process, KIs also provide information about the typology of the hotspots and seasons and hours of operation. The number of interviews with different types of KIs is tracked to ensure a broad range of perspectives is captured. All mentioned locations are collated and deduplicated daily and checked against the program listing to determine if they are already known to the local program. A final unique hotspot list is created by consolidating data from program listing and previous mapping findings, if available.

#### Level Two Mapping Activity

In the third stage of the mapping exercise, the implementation team visits each hotspot on the list generated from the level one activity and validates the hotspot as active if it is currently operational and it is a location where FSWs congregate. The team also conducts group discussions with FSWs (ie, primary KIs) on site if more than one FSW can be mobilized. If only one FSW is available and willing to participate, a one-on-one interview is conducted instead. Detailed information was gleaned about the typology of the hotspot, the peak day of the week and peak time of the day when the majority of FSWs visit the hotspot, and the minimum and maximum number of women and FSWs of all ages who come to visit the hotspot on a normal and peak day. FSWs are also asked to list other sex work hotspots in the area, so any new locations that were missed in the level one activity will be added to the list and followed up. To account for the extent of sex worker mobility, FSWs are asked about other specific locations where they connect with clients.

**Figure 2:**
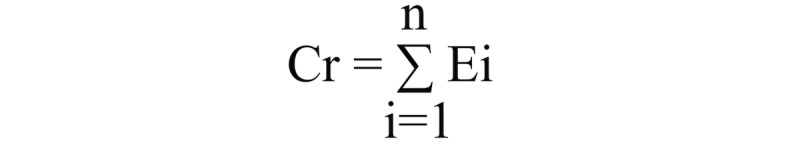
Population size estimation formula used in this study, where Cr is the crude population size estimate of female sex workers in the county; Ei is the estimated number of female sex workers in hotspot i; and i=1,2,3,...n is the number of hotspots in the county.

#### Data Analysis

Since all the identified sex work hotspots were validated during the level two activity, we used only level two data for analysis. To obtain a crude population estimate for FSWs in the entire county, the minimum and maximum estimates of all hotpots were summed and the mean was calculated. See Figure 2 for the formula used for population size estimation, where Cr is the crude population size estimate of FSWs in the county; Ei is the estimated number of FSWs in hotspot i; and i=1,2,3,…n is the number of hotspots in the county.

To account for the extent of duplication due to mobility of FSWs visiting multiple hotspots looking for clients, the following mathematical function was used: Ni = Cr(1-pi) + (Cr*pi/mi), where the adjusted estimate (Ni) is a function of the crude estimate (Cr), the proportion of FSWs who visited other hotspots (pi), and the mean number of hotspots FSWs visited (mi). Mobility was adjusted at the subcounty level; adjusted estimates for all subcounties were then summed to generate a county-wide estimate.

### Modifying the Standard Approach to Estimate the Population Size of Young Women Engaged in Transactional and Casual Sex

To answer the question whether and if so to what extent AGYW also congregate at sex work hotspots to meet casual and transactional sex partners, we built on the standard approach to programmatic mapping used for FSWs by first identifying sex work venues in Mombasa and then by asking the relevant questions about women who engaged in casual and transactional sex at these locations. Namely, we modified the premapping and planning exercise and level two interviews of the standard programmatic mapping approach but adhered to the standard practice for all other phases (Figure 1). In premapping and planning, the expanded scope of the modified method to also estimate the population size of AGYW engaging in sex work and transactional and casual sex was raised with the implementation team and peers during community engagement to prepare the team for the later phases of the mapping exercise. The study’s hypothesis about colocation of AGYW subgroups at sex work hotspots seeking different types of sexual partnership was specifically discussed. In level two interviews, the team asked primary KIs at each hotspot about the number of young women aged 14 to 24 years engaging in sex work and transactional and casual sex, the number of men who frequent the hotspot seeking FSWs and other sex partners, and other places where women visit to meet partners for transactional or casual sex.

In contrast to FSWs, mobility of women engaging in transactional and casual sex was assumed to be limited. As such, the mean peak day population estimate for women engaging in transactional and casual sex at each hotspot was taken as the best estimate for that hotspot. All hotspot-level estimates were summed to generate an estimate for the entire county.

### Implementation of the Modified Programmatic Mapping Approach in Mombasa, Kenya

The modified mapping approach was implemented between May 15, 2014, and June 20, 2014, by a 30-member team comprising International Centre for Reproductive Health Kenya (ICRH) research staff and peer educators with experience linking FSWs within their community to ICRH and other local HIV prevention and support programs. Based on existing administrative divisions, Mombasa County was segmented into 4 subcounties encompassing 9 data collection zones. The types of KIs approached for level one interviews included FSWs; men who have sex with men (including male sex workers); drug peddlers; beach boys and girls; public transport drivers (eg, taxi, taxi motorcycles, auto rickshaws, and minibuses); owners, staff, and patrons of internet shops/video dens; security guards, watchmen, and community policing groups; bouncers; bar owners, staff, and patrons; massage parlor owners and staff; brothel/sex den owners and staff; hotel/lodge owners, staff, and patrons; khat vendors; local brew sellers and patrons; staff of the Government of Kenya and NGOs; community health workers and health facility service providers; police/law enforcement agents; pharmacists; and village chiefs, assistant chiefs, and elders.

Sex work hotspots in Mombasa can be categorized into 8 general typologies: (1) public place (eg, beach, park); (2) street; (3) bar, nightclub, casino, and hotel (ie, venues with rooms); (4) bar, restaurant, and café (ie, venues without rooms); (5) guesthouse and lodge (ie, venues without bars); 6) sex den/brothel; (7) local brew den (ie, street kiosks selling *mnazi*, palm wine made from naturally fermented coconut tree sap); and (8) other (eg, home, massage parlors, saunas, video dens, and truck stops). These typology categories were developed in partnership with ICRH.

### Statistical Analysis

Chi-square test was used to compare the distribution of sex work hotspots by subcounties and the distribution of previously known and newly identified hotspots by typology. A difference is considered significant if *P*<.05.

### Ethical Approval

This study was approved in Kenya by the Kenyatta National Hospital/University of Nairobi Ethics and Research Committee; the Research Permit Committee of the National Commission for Science, Technology, and Innovation; and in Canada by the Human Research Ethics Board at the University of Manitoba.

## Results

### Population Size Estimates

We estimated that there were 11,777 FSWs (range 9265 to 14,290) in Mombasa in 2014 among whom 6127 (range 4793 to 7462) (52.03%) were between 14 and 24 years and represented about 4.54% (6127/134,885) of the general female population of the same age group. The population estimates for women aged 14 to 24 years who engaged in transactional and casual sex and congregated at the hotspots were 5348 (range 4185 to 6510) and 4160 (range 3194 to 5125), respectively. Combined, young women who engaged in transactional and casual sex represented about 7.05% (9508/134,885) of the female population of the same age group (Table 1).

### Sexual Network Overlap

**Figure 3:**
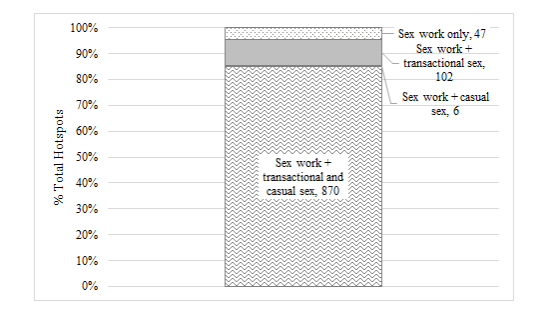
Overlap in the 1025 sex work hotspots in Mombasa County, Kenya, that were also visited by women engaging in transactional and/or casual sex.

A total of 1183 sex work hotspots were named during level one interviews, of which 1025 (86.64%) were validated to be active. Among these validated hotspots, 102 (9.95%), 6 (0.59%), and 870 (84.88%) were also locations visited by women engaged in transactional sex, casual sex, or both, respectively. Only 47 (4.58%) hotspots were exclusive sex work locations (Figure 3).

### Hotspot Typology and Distribution

Of the 1025 sex work hotspots, local brew dens were the most numerous overall, followed by bars/restaurants/cafés (Table 2).

**Table 1:** Estimated population size of women engaging in sex work, transactional sex, and casual sex in Mombasa County, Kenya.

Women engaging in:		Point estimate	Range	Proportion of general female population of the same age group, %
**Sex work**				
	All ages	11,777	9265-14,290	3.69^a^
	14-24 years	6127	4793-7462	4.54
**Transactional sex**				
	14-24 years	5348	4185-6510	4.54
**Casual sex**				
	14-24 years	4160	3194-5125	3.08

^a^Proportion of general female population of reproductive age: 319,032 (14 to 44 years).

**Table 2:** Distribution of sex work hotspots by typology and subcounty in Mombasa County, Kenya.

Sex work hotspot typology	Subcounty A^a^ (n=280), n (%)	Subcounty B^a^ (n=164), n (%)	Subcounty C^a^ (n=466), n (%)	Subcounty D^a^ (n=115), n (%)	Mombasa County (N=1025), n (%)
Public place	12 (4.3)	6 (3.7)	13 (2.8)	2 (1.7)	33 (3.2)
Street	21 (7.5)	29 (17.7)	31 (6.7)	2 (1.7)	83 (8.1)
Bar/nightclub/casino/hotel (with rooms)	63 (22.5)	40 (24.4)	81 (17.4)	26 (22.6)	210 (20.5)
Bar/restaurant/café (without rooms)	82 (29.3)	49 (29.9)	118 (25.3)	22 (19.1)	271 (26.4)
Guesthouse/lodge (without bars)	19 (6.8)	10 (6.1)	38 (8.2)	6 (5.2)	73 (7.1)
Sex den/brothel	8 (2.9)	6 (3.7)	2 (0.4)	0 (0.0)	16 (1.6)
Local brew den	68 (24.3)	19 (11.6)	173 (37.1)	57 (49.6)	317 (30.9)
Other^b^	7 (2.5)	5 (3.0)	10 (2.1)	0 (0.0)	22 (2.1)

^a^The distribution of hotspots between the four subcounties was significantly different (*P*<.001).

^b^The Other category includes hotspot types such as home, massage parlor/sauna, video den, and truck stop.

Of these 1025 hotspots, 280 (27.32%) were located in subcounty A, 164 (16.00%) in subcounty B, 466 (45.46%) in subcounty C, and 115 (11.22%) in subcounty D. The distribution of the different types of hotspots were significantly different between the four subcounties (Table 2, *P*<.001).

**Figure 4:**
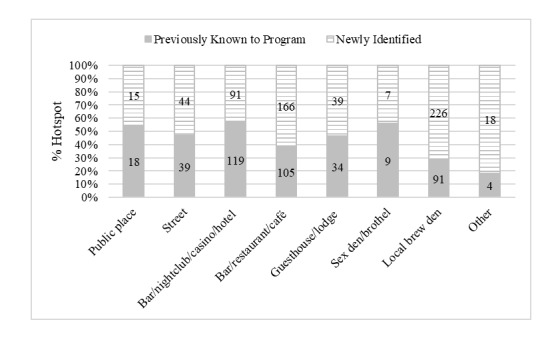
Proportion of sex work hotspots within each typology that were known to the local HIV prevention program prior to this study and those that were newly identified in Mombasa County, Kenya. The Other category includes hotspot types such as home, massage parlors/saunas, video dens, and truck stops.

Figure 4 shows the typological distribution of sex work hotspots that were already known to the local HIV prevention program prior to this study and those that were newly identified. Of the 1025 validated active hotspots, 419 (40.88%) were already known to the program and 606 (59.12%) were newly identified. The typological distribution of previously known hotspots and newly identified hotspots varied significantly (*P*<.001). Bars/nightclubs/casinos/hotels were the most common among previously known hotspots, whereas local brew dens were the most common among newly identified hotspots.

## Discussion

### Principal Findings

Using an established mapping method as the foundation, we developed a more nuanced approach to identify the distribution and population size of subgroups at risk of HIV. Our subgroups of interest were AGYW engaged in a spectrum of sexual partnerships who met their sex partners at sex work hotspots. We estimated that the 2014 FSW population size in Mombasa was 11,777 (range 9265 to 14,290), and our estimate largely falls within the range of the estimates from the 2012 mapping round using the standard approach outlined in this paper (point estimate 9288; range 6917 to 11,660) [5,24]. We also estimated that half of the FSW population was between the ages of 14 and 24 years, while a further 9500 young women in the same age group practiced transactional and casual sex. We found that there was a substantial overlap in spaces where young women met sex work clients and partners for transactional and casual sex, thereby providing opportunities for women engaging in transactional and casual sex to interact with higher risk social and sexual networks. Using this modified mapping approach, our results show that local HIV programs can use a sex work hotspot–based mechanism, as opposed to a general population approach, to reach and deliver services to AGYW subgroups at increased risk of HIV. Moreover, this modified mapping approach provides a deeper layer of strategic information for HIV/STI service design and planning and for developing representative sampling frames for surveillance and research studies.

We found that the hotspot typology profile varies significantly geographically between the four subcounties. When comparing the typologies of sex work hotspots known to the local program prior to this study and those newly identified, we additionally found that hotspot turnover was high (ie, high rates of hotspot closure and opening of new ones). Because Mombasa is a vacation destination for both Kenyan and foreign tourists alike, the vibrancy of associated industries, including the sex industry, is directly affected by the boom and slump of tourism at large [25,26]. Aside from broad economic factors, social and political pressure can also affect the viability of sex work–related businesses. Whether they are exclusive to sex work or not, businesses may suffer or may no longer exist as a sex work hotspot if there have been police crackdowns or if their popularity has dwindled among sex work clients. In both scenarios, activities between sex workers and their clients will be displaced to other locations. We have seen substantial changes among local brew dens as well as other sex work hotspots over time. Specifically, we identified a total of 317 local brew dens in 2014, among which 226 (71.3%) were new. Besides an evident increase in number, because roadside local brew dens are highly mobile, the same business can move to more desirable neighborhoods where the demand for their product is high. As a result, sex workers and their clients also tend to move with the local brew dens, in which case the new location would be registered as a new sex work hotspot in the 2014 mapping round. Furthermore, we validated 1025 active sex work hotspot in this study, whereas the 2012 mapping exercise identified only 774. This overall high hotspot turnover suggests that a significant proportion of active hotspots were not known to the program and therefore no outreach was provided to women at these hotspots. Taken together, the geographic heterogeneity in hotspot typology, high hotspot turnover, and notable increase in the number of hotspots suggest that mapping should be performed iteratively and updated over time in order for local HIV prevention programs to make the necessary service linkage and delivery adaptations responsive to the changing landscape of sex work. Recognizing the resource and time demands on local HIV prevention programs to conduct a full-scale programmatic mapping exercise, regular updates on hotspots and population size estimates can be performed by concentrating effort and resources on the level two activity during which hotspots are validated and primary KIs are interviewed to identify other nearby sex work locations. All locations new to the program should be visited and validated. This validation process needs not be one-off or intensive, but rather it can be built into the program as part of a routine practice during visits by peer educators and outreach workers to hotspots in the community to connect with members of the key population. This method has been used by the local HIV prevention program in Karnataka State, South India, implemented by Karnataka Health Promotion Trust and the Karnataka State AIDS Prevention Society. Since the initial mapping and population size estimation of FSWs and men who have sex with men in Karnataka State in 2004, the program has performed regular annual updates based on the number of key population members who were contacted by outreach and reached for services. If comprehensive mapping information is not available as the foundation, the initial hotspot list can be based upon existing program knowledge (ie, the list of locations to where programs provide outreach services). It is important to note that an estimate given for any hotspot is specific to that hotspot, whereas the size estimate of a key population in a greater geographic area (eg, town, district, county) has accounted for the movement of that key population. Therefore, while a program’s capacity to conduct comprehensive mapping may be limited, keeping track of hotspot locations and estimating key population size at a basic level would still be informative for service planning and delivery from a program’s perspective.

Programmatic mapping is a population size estimation method that is based on the collective knowledge of individuals associated with a key population or who are members themselves about where the key population of interest is located within a defined geographic area. Unlike many other population size estimation methods that generate estimates that can be wildly variable and difficult to validate [2,3], by tethering population size estimates to spaces where vulnerability and risk cluster, mapping can provide programs with reasonable estimates that can be refined as a program is rolled out and monitored over time. By overlaying data regarding HIV prevalence, sexual network, and risk behavior on top of the key population size data linked to space, this clustering of multiple factors that contribute to HIV transmission risk will help local HIV programs make strategic decisions related to geographic allocation of prevention resources, design the right intervention mix appropriate for the level of risk, and reach key populations for delivery of services to ensure program coverage and efficiency [27].

### Limitations

There are limitations to the use of programmatic mapping for identifying hotspots and estimating key population size. Because mapping involves collection of empirical data that are often sensitive, the quality, accuracy, and comprehensiveness of the data are highly dependent on connecting with informants who are knowledgeable about the structure and operation of sex work. Therefore, during the premapping phase, it is important to dedicate time, as part of project planning, to engage with the FSW community and NGOs serving FSWs in order to understand the context of sex work and adapt the mapping approach that fits with the situation. For the purposes of this study, we worked with a well-established NGO and engaged a trusted network of peer educators.

Despite a well-planned and well-executed mapping exercise, the population size estimates generated by this method still likely underestimate the true population size due to the method’s limitation to account for populations who congregate in private and virtual spaces. With the growing popularity of social networking websites and mobile apps, virtual spaces have opened up new communication channels for people to connect for sexual activities. To begin to address this question, our team has piloted a new mapping method in conjunction with analysis of internet-based networks to generate crude size estimates for online key populations [28].

Pertinent to the modifications introduced in this study to the standard programmatic mapping approach, another limitation is the accuracy in estimating the number and age of AGYW who might be at hotspots connecting with potential partners for transactional and casual sex. More work, including another round of mapping, will need to be done to validate the current estimates and refine the modified method. Nevertheless, these populations size estimates for AGYW provide a starting place for HIV prevention programs focusing on AGYW to assess the coverage of their current services and inform program adjustments as required.

### Conclusions

Programmatic mapping is an effective method that can be adapted to understand the size and geographical organization of diverse priority populations for resource allocation, program planning, service delivery, and monitoring and evaluation. Mapping has been extensively used for planning HIV/STI prevention and control programs with key populations. By adapting the standard programmatic mapping approach used traditionally for key populations, our results show that HIV prevention programs tailored to AGYW can focus delivery of interventions to traditional sex work hotspots to reach subgroups that may be at increased risk for HIV. Effective HIV prevention and control programs need to be responsive to the evolutions of an HIV epidemic, and mapping is an integral tool for the iterative process of program planning, adaptation, and evaluation.

## Abbreviations

AGYW: adolescent girls and young women
CIHR: Canadian Institutes for Health Research
FSW: female sex worker
ICRH: International Centre for Reproductive Health Kenya
KI: key informant
NGO: nongovernmental organization
STI: sexually transmitted infection
